# The implications of ritual practices and ritual plant uses on nature conservation: a case study among the Naxi in Yunnan Province, Southwest China

**DOI:** 10.1186/s13002-017-0186-3

**Published:** 2017-10-25

**Authors:** Yanfei Geng, Guoxiong Hu, Sailesh Ranjitkar, Yinxian Shi, Yu Zhang, Yuhua Wang

**Affiliations:** 1 Key Laboratory of Economic Plants and Biotechnology, Kunming Institute of Botany, Chinese Academy of Sciences, Kunming, 650201 China; 2 University of Chinese Academy of Sciences, Beijing, 100049 China; 3 Institute of Ecology and Geobotany, Yunnan University, Kunming, 650091 China; 4 CAAS-ICRAF Joint Lab on Agroforestry and Sustainable Animal Husbandry, World Agroforestry Centre, East and Central Asia, Beijing, 100193 China; 5 College of Life Sciences, Guizhou University, Guiyang, 550025 China; 6 World Agroforestry Centre East and Central Asia, Kunming, 650201 China

**Keywords:** Religious beliefs, Resource conservation, Ritual plants, The Naxi

## Abstract

**Background:**

Conservation of biodiversity is primary important of today’s critically vulnerable environment. Efficient conservation can be possible only with the long-term participation and understanding of the communities. Ritual beliefs of the indigenous people are one of the important tools to understand the local communities and aid the nature conservation. In this paper, we documented contemporary ritual practices and ritual plant uses among the Naxi people and discussed the importance of traditional knowledge on ritual practice in the conservation of plants in the mountains presenting a case study of the Dongba culture.

**Methods:**

This study was carried out from July in 2013 to July in 2014. To document and analyze the present state of the ritual plant used by the Naxi people we conducted an ethnobotanical survey. We interviewed local people including Dongba priests using the semi-structured questionnaire. During the field study, we participated in the local religious activities to witness the use of different plants in ritual activities of the Naxi people. We interviewed twenty-two key informants and eleven of them were male. All the specimens of documented species were collected and deposited at the herbarium of Kunming Institute of Botany.

**Results:**

The survey results revealed the Naxi people possessed sound knowledge of the traditional ritual plants and great diversity of plants used in many of Naxi rituals and festivals. From the survey, we documented 32 ritual plant species belonging to 24 genera of 17 families used in various ritual activities. The ritual plants were grouped into two categories, namely those burned as incense, and those used for decoration. The incense plants like *Olea europaea* subsp*. cuspidata* and *Pistacia weinmanniifolia* were probably promising natural aromatic resource. Plants of genus *Quercus* were the most frequently used species. The places for ritual activities were diverse, such as the incense burners inside and outside the house and sacred trees at the Baishuitai. Local people except the young generation had an abundant of traditional knowledge.

**Conclusions:**

Our study shows the live ritual activities and the beliefs of the residents are keeping the plant diversity and the entire forest preserved as sacred mountains. Our study emphasizes traditional belief and an alternative view of conservation that is not led mainly by governmental policies, as local practices and ritual plants uses play as constant reminders to the Naxi on nature conservation. However, further research is recommended for in-depth understanding the role of traditional belief in biodiversity conservation.

## Background

In the Anthropocene epoch, human population growth and related land use transformation severely affected the nature. Alteration of habitats and associated biological changes threatened the existence of important species as well as the ecosystem. Still plants from the natural sources are very significant to a larger number of human population. In the mountains, plants are the important source of energy as food, a construction material to build houses, a main ingredient of the health care. In addition to these century-old uses, until today several plants are part of various ritual purposes [1], as well as a source of livelihood for the local people [2]. Conservation of such an important diversity is the primary concern of the today’s critically vulnerable environment. Efficient conservation can be possible only when the technical expertise is combined with an understanding and consideration of the cultural practices of local communities [3, 4]. Ritual beliefs of the indigenous people are one of a prominent tool to understand the local communities and aid the nature conservation. Many aboriginal communities preserve their tradition through folklore and follow ritual beliefs [1], which can provide valuable information and link to the conservation of biodiversity.

Conserving biodiversity based on cultural and religious faiths is often more efficient and sustainable than based only on governmental legislation or regulation [5]. The aboriginal community believes that ritual plants can be applied to ritual healing [6, 7] and used as incense or ornaments for the communications with spirits [8]. In China, a long history incense use for sanitation, religious activities, insecticidal and warfare exists, such uses are common in Confucianism, Daoism and Buddhism [9]. Throughout the world many ethnic groups follow their ritual beliefs and use plant diversity for ritual purposes. Ritual uses of plants might be related to religious or other activities. An ethnic group called the Naxi in the Northwest part of Yunnan province of China uses plenty of ritual plants during festivals for repayment for nature. Such festivals provide the Naxi opportunities to come together and celebrate the rituals followed the ancestors since a long time. Incense burning and other ritual activities are main parts of Naxi festivals.

**Fig. 1:**
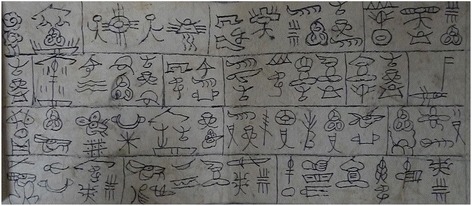
First page of one Dongba classics written in pictographs used on the Heaven Worshipping Day

The Naxi is one of the most ancient peoples and well known worldwide because of their unique Dongba Culture. The Dongba culture refers to the total of the Naxi traditions, customs, beliefs and ways of life. The Naxi language is a part of Sino-Tibetan language family, Tibeto-Burman branch and follows two main dialects. The Naxi developed a pictographic script which is the only script still in use around the world. It is primarily utilized by the Dongba priests when conducting ceremonies and rituals [10] (Fig. 1). Dongba priests are wise artists and are central to Naxi cultural life [11]. The Naxi believes in the spiritual existence of mountains and rivers, trees and herbs, animals and humans. The Shu, which is thought to be the spirits of nature, is the most important. The Naxi myth explains farmland and livestocks are part of the human realm and the mountains and the rivers belong to Shu. At one time, there was a dispute between human beings and the Shu due to the human’s invasion into the territory of Shu. The Myth tells Dongba priest played a role of the mediator and regained the harmony between the human and the Shu. According to the treaty, humans need to worship the Shu as the god of nature every year, in turn, Shu must provide human need from nature and stop assaulting them [12]. The religious activities of the Naxi are focused on regulating the relationship of spirits with the live descendants. They burn incense and offer sacrifices to repay nature spirits for the damage done, such as hunting wild animals, cutting trees, and so on.

Thus, we aim to document and analyze the current status of ritual practices and ritual plant uses by the Naxi during their ritual activities. The relevant knowledge on ethnobotanical practices and beliefs can be supportive material to discuss how religious beliefs can contribute to conservation.

## Methods

### Study area

**Fig. 2:**
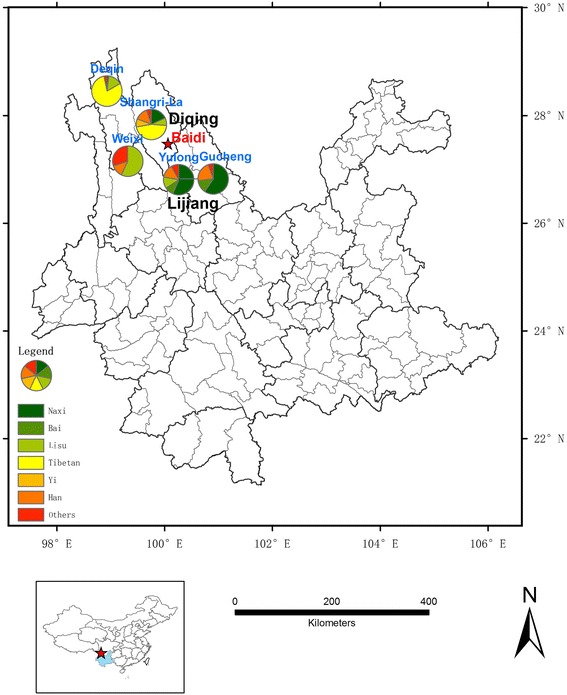
The location of Baidi village and main minorities nearby in Northwest Yunnan, China

The study area covers a small village called Baidi village in the Northwest part of Yunnan Province of China. The geographical location of Baidi Village (Sanba Naxi Nationality Township, Shangri-La City, Diqing Prefecture) is between northern latitudes of 27°30′ and 27°28′, and the eastern longitudes of 100°01′ and 100°05′ (Fig. 2). The Naxi population is mainly residing in Lijiang city, and the Naxi in Baidi is often considered the purest of their race [12]. Main minorities include Tibetan, Naxi, Bai etc. in Diqing (Weixi county, Deqin county and Shangri-La city) and Lijiang (Gucheng & Yulong only) are also illustrated (Fig. 2).

Baidi-Naxi obeys their old religious customs that combine the shamanism and the pre-Buddhist, Bon religion of Tibet [12, 13]. Baidi comprises approximately 3000 inhabitants and 75% of them are the Naxi. Eight Dongba priests evenly exist in eight small communities of Baidi village to perform ceremonies for the villagers.

We conducted intensive fieldwork in the Baidi village from July 2013 to July 2014. Field study included semi-structured interviews, field observations, participation in the ritual activities and in-home observations. We interviewed 22 key informants, including two Dongba priests, chosen based on the knowledge of common traditions and religious beliefs. The age of informants ranged from 25 to 86, and the sex ratio was 1:1. The interviews covered the questions on the ritual plant uses, related beliefs, knowledge and practices (Appendix 1). We prepared a standard format for documenting ritual plants. It included the informant’s details, species name, part used and other specific purposes. We collected all listed plants and identified voucher specimens following the nomenclature of the Flora of China [14], and they were deposited at the herbarium of Kunming Institute of Botany (KUN).

## Results

### Ritual plants and incense culture in Baidi

We observed the wide variety of plants used in many of Naxi rituals and festivals. The survey results revealed the Naxi people possessed sound knowledge of the traditional ritual plants. Branches and flowers of 32 plant species belonged to 24 genera of 17 families and were very common in the study site (Table 1). The most commonly used plants were from the family Fagaceae followed by Rosaceae and Pinaceae. The ritual plants can be grouped into two categories, burned as incense, and used for decorating incense table, suspending on the doors and wearing on the human body. However, some plants have multi-purpose uses; for example, *Quercus pannosa* can be burned as incense, suspended on the doors and worn on the head.

**Table 1:** Ritual plants used by the Naxi in Baidi, Northwest Yunnan province, China

Order	Scientific name	Family	cLocal name	Habit	Usage	Parts used	Use frequency	Specimen number
1	*Acorus calamus* L.	Acoraceae	changpu	Herb	on many occasions, for decorating incense table and suspending on the door	leaves	***	PHO1408
2	*Pistacia weinmanniifolia* J. Poisson ex Franchet	Anacardiaceae	yizhu	Shrub	on many occasions, for burning and suspending on the door	branch	***	GH55
3	*Artemisia argyi* H. Lév. & Vaniot	Asteraceae	bu	Herb	on many occasions, for burning and suspending on the door	branch	***	GH93
4	*Artemisia carvifolia* Buch.-Ham. ex Roxb.	Asteraceae	haozhi	Herb	on many occasions, for suspending on the door	branch	***	GH134
5	*Hippolytia delavayi* (Franch. ex W. W. Smith) Shih	Asteraceae	buna	Herb	on many occasions, for suspending on the door and tied to the body on Duanwujie	branch	***	GH114
6	*Cephalotaxus fortunei* Hook.	Cephalotaxaceae	shanshu	Shrub or Dungarunga	on many occasions, for burning and suspending on the door	branch	***	PHO1495
7	*Hypericum forrestii* (Chittenden) N. Robson	Clusiaceae	muwanni	Shrub	on Duanwujie (Dragon Boat Festival), for decorating incense table	branch, flower	**	GH174
8	*Cornus officinalis* Sieb. & Zucc.	Cornaceae	amudagu	Shrub or Dungarunga	on many occasions, for burning and suspending on the door	branch	**	GH276
9	*Dendrobenthamia capitata* (Wall.) Hutch	Cornaceae	laka	Shrub or Dungarunga	on many occasions, for burning and suspending on the door	branch, flower	***	GH86
10	*Juniperus chinensis* L.	Cupressaceae	bashu	Tree	on many occasions, for burning and suspending on the door	branch	***	PHO1493
11	*Rhododendron decorum* Franch.	Ericaceae	niziba	Shrub	on many occasions, for burning and suspending on the door	branch, flower	***	GH119
12	*Campylotropis polyantha* (Franch.) Schindl.	Fabaceae	ba	Shrub	on 7th February (the Heaven Worshipping Ceremony), for decorating heaven-worshipping altar and headwaters	branch, flower	*	GH142
13	*Quercus aliena* Blume	Fagaceae	laba	Shrub or Dungarunga	on many occasions, for burning, suspending on the door and sticking into the hair	branch	****	GH99
14	*Quercus aquifolioides* Rehd. & E. H. Wils.	Fagaceae	laba	Shrub or Dungarunga	on many occasions, for burning, suspending on the door and sticking into the hair	branch	****	GH62
15	*Quercus cocciferoides* Hand.-Mazz.	Fagaceae	laba	Shrub or Dungarunga	on many occasions, for burning, suspending on the door and sticking into the hair	branch	****	GH188
16	*Quercus fimbriata* Chun & Huang	Fagaceae	laba	Shrub or Dungarunga	on many occasions, for burning, suspending on the door and sticking into the hair	branch	****	GH186
17	*Quercus pannosa* Hand.-Mazz.	Fagaceae	laba	Shrub or Dungarunga	on many occasions, for burning, suspending on the door and sticking into the hair	branch	****	GH269
18	*Quercus* sp1	Fagaceae	laba	Shrub or Dungarunga	on many occasions, for burning, suspending on the door and sticking into the hair	branch	****	GH98
19	*Quercus* sp2	Fagaceae	laba	Shrub or Dungarunga	on many occasions, for burning, suspending on the door and sticking into the hair	branch	****	GH185
20	*Punica granatum* L.	Lythraceae	shiliu	Dungarunga	on many occasions, for decorating incense table	branch, flower	**	PHO1490
21	*Myrsine africana* L.	Myrsinaceae	azabagong	Shrub	for burning	branch	*	GH76
22	*Olea europaea* L. subsp. *cuspidata* (Wall. ex G. Don) Cif.	Oleaceae	xuge	Shrub or Dungarunga	for making joss stick by a few people	branch, flower	***	GH112
23	*Picea likiangensis* (Franch.) E. Pritz.	Pinaceae	shanshu	Tree	on many occasions, for burning and suspending on the door	branch	***	PHO1492
24	*Pinus armandii* Franch.	Pinaceae	situobei	Tree	on many occasions, for burning and suspending on the door	branch	***	GH250
25	*Pinus yunnanensis* Franch.	Pinaceae	situobei	Tree	on many occasions, for burning and suspending on the door	branch	***	GH356
26	*Tsuga chinensis* (Franch.) Pritz.	Pinaceae	shanshu	Tree	on many occasions, for burning and suspending on the door	branch	***	PHO1494
27	*Hordeum vulgare* L.	Poaceae	mai	Herb	on 7th and 8th February, for spreading on the burning plants	seed powder	*	GH146
28	*Amygdalus persica* L.	Rosaceae	buji	Dungarunga	on 7th February (the Heaven Worshipping Ceremony); for decorating heaven-worshipping altar and headwaters	branch, flower	*	GH170
29	*Armeniaca vulgaris* Lam.	Rosaceae	buka	Dungarunga	on 7th February (the Heaven Worshipping Ceremony), for decorating heaven-worshipping altar and headwaters	branch, flower	*	PHO1491
30	*Philadelphus delavayi* L. Henry	Rosaceae	saka	Dungarunga	on 7th February (the Heaven Worshipping Ceremony), for decorating heaven-worshipping atar and headwaters	branch, flower	*	PHO1497
31	*Pyrus pyrifolia* (Burm.f.) Nakai	Rosaceae	lishu	Dungarunga	on 7th February (the Heaven Worshipping Ceremony), for decorating heaven-worshipping altar and headwaters	branch, flower	*	PHO1410
32	*Leptodermis* sp	Rubiaceae	ba	Shrub	on 7th February (the Heaven Worshipping Ceremony), for decorating heaven-worshipping altar and headwaters	branch, flower	*	PHO1494

Species in inventory are arranged firstly by family taxa and then by genus taxa. Stars * in Row Use frequency represent the use intensity, and more *, more preferred. Voucher number with PHO means voucher photograph number, and the one without PHO means voucher specimen number

Around 67% of 32 ritual plant species are used as incense. Every month people burned incense on the first and fifteenth day and during local festivals. In addition, the Baidi-Naxi burned incense on many occasions, during out-migration; when people get sick or have a bad dream; the demise of a family member, children taking exams and New Year. Besides, every day was designated as one of 12 zodiac signs by the Naxi and they burn incense accordingly. For example, 16th July 2014 (solar calendar) was a tiger day, on that date, those born within the year of the tiger burn incense. On Eryueba, the incense burning was most magnificent and collective activity.

**Fig. 3:**
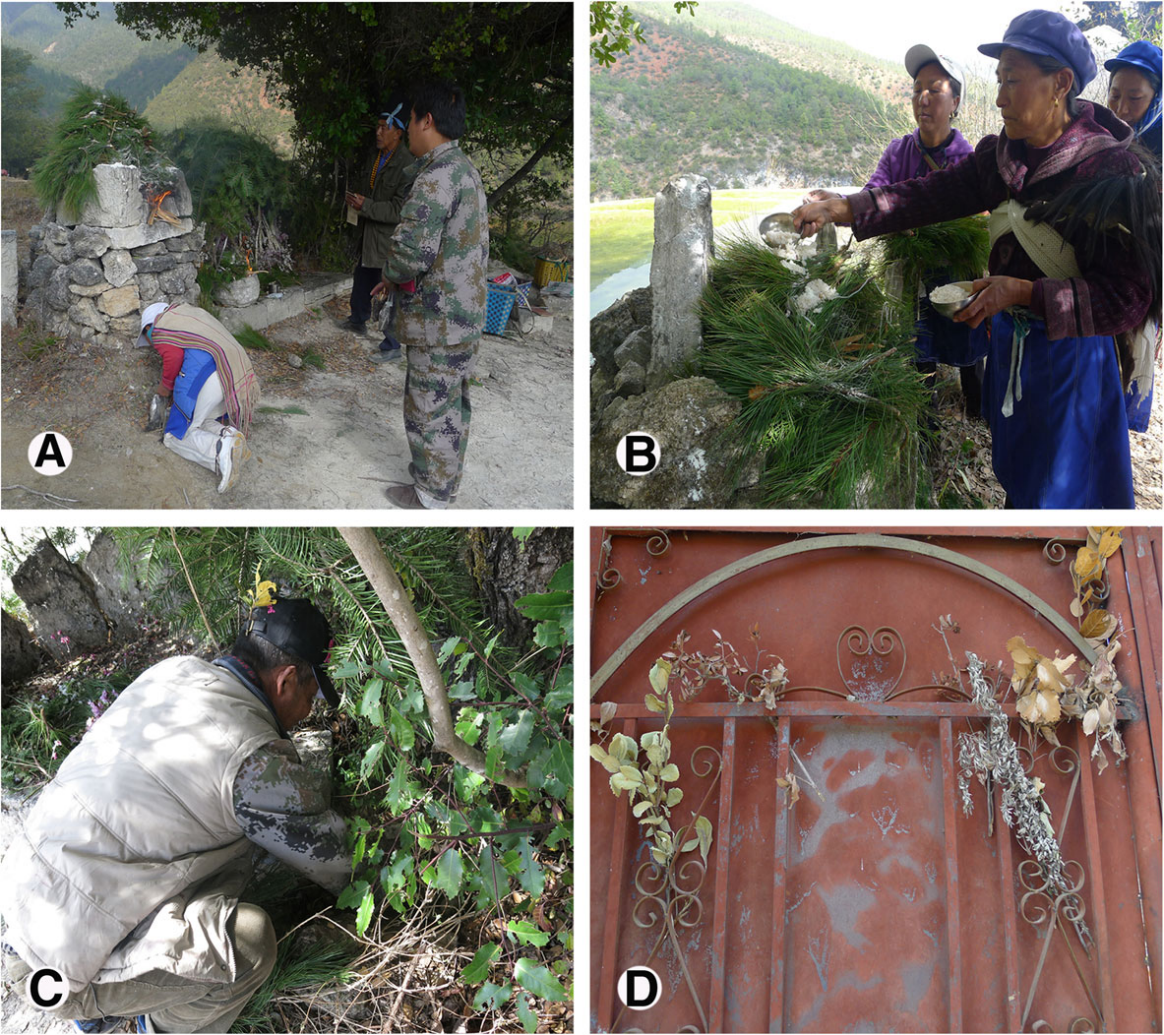
Naxi Worshiping and ritual plants use activities. **a** A Naxi woman is worshipping to the Heaven. **b** The Naxi put boiled rice on the incense plants before burning. **c** Leaves of *Quercus* were stuck in the hair by the Naxi. **d** Ritual plants used for suspending on the doors

While burning incense in Eryueqi, the Dongba priest chanted the Dongba classic spells (Fig. 3a). Usually, the cooked rice or flour was put along the burning incense (Fig. 3b) as a repayment to the nature spirits for taking away the natural resources. According to the local residents, *Juniperus chinensis*, *Olea europaea* subsp*. cuspidata,* and *Pistacia weinmanniifolia* were the three species that most highlighted by the local people and smelled most aromatic to their senses. People used the leaves of *O. europaea* subsp. *cuspidata* to make joss sticks in the past.

Except those burned as incense, another category of the ritual plants use were for decoration. *Quercus* species were kept on the doors and worn on the head except burning (Fig. 3c). People believe such activities repel diseases causing spirits and evil intentions of unwanted spirits. *Hypericum forrestii* and *Acorus gramineus* were used to decorate the incense table during Duanwujie. *A. gramineus* and *Artemisia argyi* also were worn on the head and waist during Duanwujie. People also kept *A. argyi*, *A. carvifolia* and *A. gramineus* on the doors to ward off bad luck and the bad spirit (Fig. 3d). In this study, we found species of genus *Quercus* as the most frequently-used genus for ritual purpose.

### Ritual times and places

**Fig. 4:**
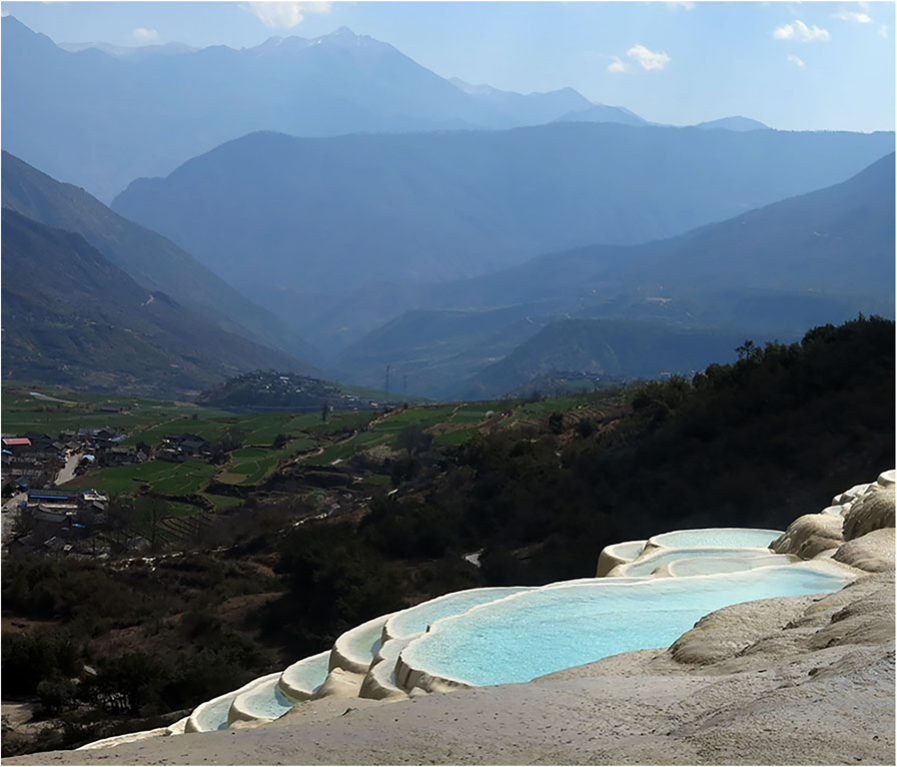
The view of Baishuitai-one of the biggest the limestone terrace in China

The Naxi festival represents the art of Naxi living. The Naxi year follows a rhythm of festivals and religious observances, ranging from solemn family gatherings to national celebrations at the Baishuitai. The ‘Baishuitai’ is a big limestone terrace meaning white water terrace in Chinese (Fig. 4) and regarded as ‘Holy Land’. According to the Naxi Myth, the Naxi ancestors learned the Terrace Cropping systems from the shape the Baishuitai. It is a natural site chosen by the people since time immemorial for performing the religious activities. Residents strictly protected the natural resources in such sacred land. Every object at the Baishuitai considered as divine, especially the plants. People cannot harvest any plants except few tree branches and flowers for religious purposes, portraying local belief supporting conservation of nature.

Throughout the year, villagers celebrate countless festivals, and some of the locally available plants are a crucial part of such rituals. Dongba priests perform most of the ceremonies in local festivals, guided by their pictographic ritual books. Spring is the time of year of the majority of festivals (Appendix 2), when the Naxi sows and hunts, as well as performs elaborate rites of passage surrounding birth and death. Most festivals are hosted according to the Chinese lunar calendar. The most important times during the lunar month are the first day and the fourteenth or fifteenth day.

**Fig. 5:**
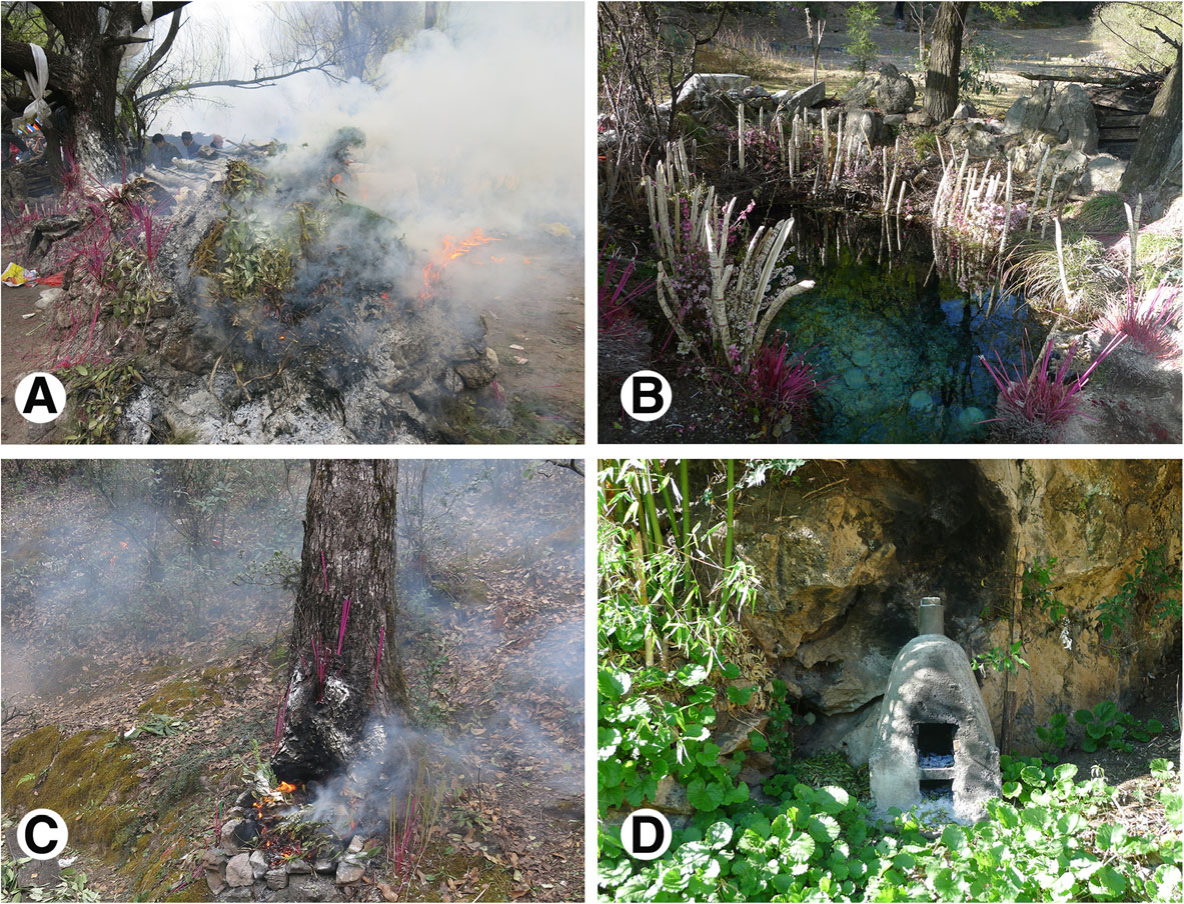
Incense burning activities and various places for worshiping. **a** Incense burning at the biggest incense burner on Eryueba. **b** Flower and peach wood used as decorations for the Dragon Pool on Eryueqi ceremony. **c** Incense burning at the Holy Tree on Eryueba. **d** The nearby incense burners for small ceremonies in every community

Two biggest ceremonies for the Naxi in Baidi were the Eryueqi and Eryueba celebrated on 7th and 8th of the second month of a year respectively, according to the lunar calendar. The both ceremony were performed at the Baishuitai. Eryueba is the most important festival devoted to the Shu nature spirits. Everyone in Baidi and some Naxi from Lijiang visited the Baishuitai just after their breakfast to celebrate the great festival. Every family burns their incense as soon as they arrive at Baishuitai in the morning of Eryueba (Fig. 5a). The burning plants are small branches of ritual plants the Naxi regarded as sacred ones. Eryueqi, also called the Heaven Worshiping Ceremony, is a grand festival and celebrated by only one community of Baidi due to historical reasons. During Eryueqi, people collected plants (flower and peach wood) from Baishuitai for decorating the Dragon Pool (Fig. 5b). The Dragon Pool is regarded as purity and receives water from the natural source. After worshiping, people brought a bottle of water back home as holy water. Activities performed during two major festivals represent act to repay the nature.

In our study, one of the most important uses of the ritual plant was incense burning to worship deities. In every community, people made incense burners. The trees at the Baishuitai also served as a place for joss sticks and incense plants burning (Fig. 5c). Every family has their fixed Holy Tree at the Baishuitai for ritual ceremony every year. They will put out the incense burning carefully after the ritual ceremony. We observe different community use these specific places in the respective communities to burn incense. Residents closer to the Baishuitai visit it accordingly to burn incense, whereas those residing far went to Baishuitai only for ritual activities on major festivals. People burn incense inside the house or in the incense burner near the houses (Fig. 5d). Elder members of the community used to burn incense every day at the Baishuitai during their zodiac year.

### Insight into the dynamics of traditional beliefs, cultural-tourism and local development

Elder and mid-age Baidi-Naxi possessed rich knowledge on ritual plants and believed in ritual practice. However, young people knew little about ritual plant uses and practices. We have documented the interaction between a 15-year-old girl before attending high school entrance examination and her grandmother. Grandmother burned incense at the Baishuitai for young girl’s success. The girl reacted differently to grandmother “it is useless, why you are still doing this? Even if you perform such rituals, it won’t change anything. I will get a good score based on what I learned”. This conversation was a simple example. We found the dramatic decrease on the traditional belief among the young Naxi, which Naxi villagers thought conserving nature until today.

The religious beliefs and traditional practices among the Naxi in Baidi are animistic in nature. We observed the cultural and esthetic value of this village attracts thousands of tourists from different parts of China and the world. Most of tourists were interested in participating the local festivals. Baidi-Naxi generated a lot of revenue from charging tourists since Baishuitai was declared as a scenery spot in 1988. Local villagers enjoyed a dividend for providing services to tourists like guiding the horses as means of travel and cleaning services in Baishuitai. Local hotels and restaurants also showed a booming trend in recent years because of increasing number of tourists.

## Discussion

Our results show some plant species are important for Baidi-Naxi to perform ritual practices. Such plant uses deeply rooted in the beliefs of the indigenous Naxi. Local festivals and ritual plants used are constant reminders to the Naxi that people must control greed and take from nature only to satisfy a basic need. The belief and living philosophy of Baidi-Naxi are not unique but still many components make them distinct. In Northeast Yunnan, Bai people also use plants of family Ericaceae but different plant species from the Naxi for the ritual purpose [9]. A study states that sacred trees grow near holy water sources [15], and Baidi-Naxi also have ritual trees close to the Dragon Pool at the Baishuitai. The most common function of the holy trees is to serve as the abode of the spirit [16] and the white flower around the headwaters symbolize purity [17]. The incense has had a continuous religious significance throughout the history from the first civilization to the present day. It is used to purify and to please gods and as an offering to the Heaven [18]. In traditional beliefs, ritual plants are spiritual mediums to connect human beings with invisible force [19]. These common symbols strengthen local peoples’ faith in every way and everywhere.

In many countries, sacred natural sites are the world’s oldest form of habitat conservation. Indigenous communities often share and govern such sites like the Naxi in the protection of nature [20]. Tracing the origin to the Naxi tradition of conserving sacred site is hard. The religious beliefs bonded with such a place are keys to conservation just as holy lands in India [21, 22]. The existence of a sacred natural site intensifies the traditional beliefs and vice versa. The interaction between people and environment is a matter of spiritual concern, and such religious beliefs have been called profoundly ecological [23]. Many faiths have attached importance to biodiversity, including plants and animals in or out of the sacred natural sites. In our study, *Quercus* plants are particularly thought as the cult by the Baidi-Naxi and are symbolically used everywhere in the village. Ritual plants are emotionally powerful cultural symbols in the local society, and they help to maintain a sense of respect for nature, just like the Baidi. Forests with high spiritual value can easily convert into a protected area to help its survival, e.g. the kaya forests in coastal East Africa [24, 25], this study argues that faith groups owned and managed areas may aid conserving biodiversity globally.

The world’s major culture and ritual practices observe conservation of biological diversity and nature environment as essential for human [26–29]. Similarly, the Naxi tradition, ecological belief, Holy Mountain with Dongba culture, worship and protection for sacred trees has improved the protection of biodiversity and environment [30]. The ritual practices of the communities like the Baidi-Naxi are respectful and conserving the natural resources and the ecology of natural systems [31]. In Central Himalaya, the religious philosophy of the conservation of plants has been a valuable tool to protect natural resources [32]. In Zimbabwe, traditional spiritual values have motivated the protection of some of the dry forests [33]. In Ghana, fostering strategic alliances between culture and conservation have successfully protected Sitatunga population [34]. In most of Africa, spiritual beliefs can strongly make service to resource conservation and environmental protection [35–37]. The indigenous people’s respect for religious or sacred ecological values has, to some extent, served the biodiversity conservation [38]. It is important to cry out for action between the movements of ritual beliefs and natural resource conservation all over the world [39].

However, strengthening the traditional beliefs or not has been a controversial topic [40]. Some hold that there always has a gap between belief and practice. How fast or in what social domain indigenous knowledge might be lost with modernization and to what extent the knowledge can coexist with changing values and modernization is not clear [41–43]. The complementarities of religious belief and scientific knowledge may become an increasingly important topic in resource management. Sustainable utilization of forest and associated land resources is a complex issue that encompasses societal needs, ethical and cultural values, and economic status of Baidi village. The adoption of cultural tourism as a livelihood strategy by the Naxi is the part of Naxi culture about adapting to the environment constantly. Tourism is particularly the marginal economy for individuals and is seasonal (for example, hotel business) in Baidi. Cultural tourism created directly and indirectly from Baishuitai has positively impacted local villagers’ livelihoods. In Baidi, cultural tourism also expanded the Naxi religious influence and enhanced cultural self-confidence. Thus forest resources got better protection. Dying traditional faith will cost the lost in one factor favoring the conservation of natural resources. Therefore, the younger generation should be educated on indigenous practices and culture, but precaution is necessary to separate real faith from superstitions.

## Conclusion

There is rich knowledge of local festivals and ritual plants use in Baidi. Ritual plants play a major role in Naxi people’s festivals and daily life. Our study show the live ritual activities and the beliefs of the residents are keeping the plant diversity and the entire forest in Baishuitai preserved as sacred mountains. Now religious beliefs represent a descending trend among the younger generation. We think strengthening knowledge of and respect for traditional beliefs and publicity of sacred natural sites can increase tourism. The increase in economic activity with more tourist flow and demand of mediator may motivate youngster to learn their indigenous culture. Continuation of the ritual practices has significant potential for natural resources conservation. Therefore, it is reasonable to keep alive the traditional beliefs and practices that can significantly contribute to the rural development and nature conservation. We seek to emphasize traditional belief and an alternative view of conservation that is not led mainly by governmental policies, as local practices and ritual plants uses play as constant reminders to the Naxi on nature conservation.

## Appendix 1

### Outline of a semi-structured interview

1. What is your age?
2. How many Naxi festivals do you usually have now? What is the origin of each festival? What is the main purpose of each festival?
3. What are the main activities of each festival?
4. Why do we still celebrate these festivals?
5. What kind of ritual plants do you use very often in your daily life?
6. What is the place for ritual plants?
7. What is the meaning of each ritual plant?
8. What is the Naxi name or local name of each ritual plant, is there any connection between the name and its meaning or purpose?
9. What is the common habit of each ritual plant?
10. What are the occasion and the specific usage?
11. What are common used parts?
12. How often do you use each ritual plant?
13. Do you still believe in the ritual use of the plants?
14. Do you think the Naxi belief helps the local nature conservation?
15. Are there any regulations about sacred trees or mountain?

## Appendix 2

### Important festivals in Baidi

Many festivals and ceremonies are based on the religious beliefs of the Naxi people. The ritual plant uses mainly occur in the first and the last day of every month and local festivals. Key informants were interviewed on most important festivals throughout the year. The dates in brackets follow the lunar calendar.

#### Spring Festival (01.01)

The Spring Festival is the second most important festival for the Naxi (the most important one is Eryueba, see following). On this day they always burn incense in or out the house, and go to Baishuitai for worshiping.

#### Worship to livestock spirit (01.19)

This day is to worship to the spirit who safeguards the livestock of the local people. They sacrifice the chicken to the spirit and burn incense too.

#### Jitian-Worship to the Heaven (02.07)

Only the Naxi people from Bowan community in Baidi village can attend this worshiping ceremony normally. Outsiders sometimes are allowed to participate with the permission of the Dongba priest. On this day, Dongba priest recites seven Holy Books all written in pictographic script when they carry out the ceremony as well as incense burning. Also one pig and two hens are sacrificed to the Heaven.

#### Eryueba (02.08)

This is the feast that immediately follows Eryueqi, and it is the most important festival and the ceremony to the Shu nature spirits. Everyone in Baidi and some Naxi from Lijiang go to Baishuitai to celebrate the day after breakfast. They burn incense, make sacrifice, worship the Heaven and cook at the Baishuitai. After lunch, they dance and sing together merrily. They do not go back home until sun set. On this day Dongba priest play no special role.

#### Duanwujie-Dragon Boat Festival (05.05)

Before and after the Dragon Boat Festival, the Naxi go out to pick up a yellow flower named muwaniba in Naxi language (muwani means duanwu, ba means flower, scientific name is *Hypericum forrestii*) and decorated the incense table with it. Normally, they burn incense in the morning.

#### Huobajie-The Torch Festival (06.24)

A festival is considered the small year among the Naxi common to most of the Yi people. This is also a big festival, and almost everyone in Baidi goes to Baishuitai (see Eryueba).

#### Jingjiujie-Qiyueban (07.15)

In past times, the husbands come back home from long time forest grazing and hunting on this day. So the wives, as a tradition, burn incense, sing, dance and toast to the husbands for expressing their gratitude of the husbands’ sublime toil.

#### Zhongqiujie-Middle Autumn Day (08.15)

They do not eat mooncakes that much like the Han nationality, but they do burn incense and have a family reunion on that day.

#### Chuxi (the day before 01.01)

After the fancy dinner, they burn the incense inside the house all the night. This is a time when families get together to celebrate renewal.
